# A Numerical Study on the Effect of Variable Wear Coefficient on Fretting Wear Characteristics

**DOI:** 10.3390/ma14081840

**Published:** 2021-04-08

**Authors:** Shengjie Wang, Magd Abdel Wahab

**Affiliations:** 1 Department of Electromechanical, Systems and Metal Engineering, Soete Laboratory, Faculty of Engineering and Architecture, Ghent University, 9000 Ghent, Belgium; shengjie.wang@ugent.be; 2 Division of Computational Mechanics, Ton Duc Thang University, Ho Chi Minh City 700000, Vietnam; 3 Faculty of Civil Engineering, Ton Duc Thang University, Ho Chi Minh City 700000, Vietnam

**Keywords:** fretting wear, wear models, wear coefficient, wear mechanisms, numerical modeling

## Abstract

Fretting wear is a common phenomenon that happens between contact parts when there is an oscillatory relative movement. To investigate wear characteristics history in the fretting process, the finite element method (FEM) is commonly applied to simulate the fretting by considering the wear in the model. In most literature publications, the wear coefficient is considered as a constant, which is not a real case based on the experimental results. To consider the variation of wear coefficient, a double-linear model is applied in this paper, and the tribologically transformed structure (TTS) phase is considered in the study of the wear coefficient variation model. By using these models for variable wear coefficient for both flat and cylinder, the difference of wear characteristics, plastic strain, and stress between variable wear coefficient model (VWCM) and constant wear coefficient model (CWCM) are analyzed. The results show that the variable wear coefficient has no significant effect on the wear characteristic at the end of the process in the gross sliding regime. However, in the partial slip regime, the effect of variable wear coefficient on wear characteristics is significant. Due to the difference in contact geometry in the fretting process between VWCM and CWCM, the tangential and shear stress and equivalent plastic strain also show differences during the fretting process.

## 1. Introduction

Fretting is a common phenomenon that happens between contacting parts, which is caused by oscillatory relative movements under normal load. Fretting regimes can be classified into stick regime, mixed stick-slip regime, and gross slip regime based on relative slip amplitude, which was first analyzed by Vingsbo [1]. Fretting damages can be found in the fretting process, such as wear, crack caused by fatigue and corrosion [2,3,4]. Fretting wear is a kind of surface degradation, which can be divided into three phases: metal adhesion and transfer, generation of debris, and stable state [5].

Many parameters can affect the fretting wear characterization, among which the main effect comes from slip amplitude, normal load, material properties, coefficient of friction, and geometry of the contact parts [6,7,8,9,10]. Hager et al. did an experimental investigation on the difference of fretting regimes: the relationship between friction force and displacement is a straight line in the stick regime, an elliptical shape in the partial slip regime, and quasi-rectangular shape in the gross sliding regime. In addition, the normal transition load from the partial slip regime to the gross sliding regime increases with the increment of the applied displacement amplitude. Guo et al. analyzed the normal load and sliding amplitude on wear coefficient for alloy 690 and found that the wear coefficient increases then tended to be constant with the increase of the normal load and applied displacement [11]. Moreover, the effect of debris generated in the fretting process was analyzed by many researchers [2,12,13]. The effect of wear on fatigue was also commonly investigated in current research to make a combined wear and fatigue model [14,15,16,17,18]. From the above-mentioned parameters, we can see that fretting wear is a complex phenomenon. To better analyze this phenomenon, we should distinguish the primary and secondary parameters and consider as much as we can to do a better simulation after considering both efficiency and accuracy.

The vital and persistent goal for the engineers and designers in tribology is to have robust mathematical equations to calculate the wear rates and translate the microscopic experimental results into the macroscopic model in the fretting process [19]. Both physical and chemical interactions happen between contacting mechanical components, which should be considered in the analysis of wear. To investigate the details of fretting wear, microscope experiments were conducted by many researchers [20,21,22]. For example, Zhu et al. investigated the coating effect on the coefficient of friction and wear mechanism in the whole fretting process for Al–Si alloy by SEM morphology [21]. It was concluded that the coating could reduce the coefficient of friction in the stable stage. Moreover, with the coating, the wear mechanism was delamination in the partial slip regime and a combination of delamination and abrasive wear in the gross sliding regime. From this example, we can see that for specific material and loading conditions, the wear mechanism and parameters differ greatly. Therefore, general, and practical models are essential for the accurate prediction of fretting wear. In addition, better results can be obtained if more variables are considered in the simulation.

In this paper, the effect of variation of wear coefficient during the fretting process on wear characteristics and stress is analyzed in both partial slip regime and gross sliding regime. Moreover, equivalent plastic strain is considered in the whole fretting process. This paper is structured as follows: after the introduction of fretting wear, the wear models and problem description are given in Section 2. The referenced experimental setup is described in Section 3. Then, the details of the FE model are given in Section 4, after which the results and discussion are presented in Section 5. Finally, the conclusion of this paper is reported in Section 6.

## 2. Wear Models and Problem Description

FEM is commonly used to simulate the fretting wear process due to both efficiency and accuracy [23,24,25]. The basic theory for the wear model is Archard’s equation [26]:

(1) $$V=k_{A}SP$$

where $k_{A}$ is Archard wear coefficient, S is the total sliding distance, P is the normal load, and V is the wear volume. S can be obtained by multiplying 4 times relative slip amplitude δ (half stroke) and the number of cycles, N. Then Equation (1) can be written as:

(2) $$k_{A}=\frac{V}{4\times \delta \times N\times P}$$

McColl et al. implemented Archard’s equation in their FE model and used a modified Archard’s model given by [27]:

(3) $$\Delta h_{i,j}=k_{A}\times s_{i,j}\times \Delta N\times p_{i,j}$$

where $\Delta h_{i,j}$, $s_{i,j}$, and $p_{i,j}$ are local wear depth, relative slip, and at node i in jth increment, $k_{A}$ is the Archard wear coefficient, as shown in Equation (1) and ΔN is the jump cycles, which is used to decrease the computational time. $k_{A}$ is usually considered a constant the total wear volume at the end of the test as described in Equation (2).

By analyzing the relation between dissipated energy and wear volume, a dissipated energy wear model was proposed by Fouvry et al. [19]. This model was applied to FE prediction of wear in [28], and its equation is given by:

(4) $$\Delta h_{i}\left(x\right)=k_{E}\int_{t=0}^{T}q_{i\;}\left(x\right)ds_{i}\left(x\right)$$

where $\Delta h_{i}\left(x\right)$, $q_{i\;}\left(x\right)$, and $s_{i}\left(x\right)$ are wear depth, shear stress, and relative slip in ith cycle at position x, $k_{E}$ is the dissipated energy wear coefficient and T is the period of one cycle. In Equation (4), $k_{E}$ can consider the variation of coefficient of friction μ, whereas, in Equation (3), the coefficient of friction is assumed to be a constant. The relation between Archard’s wear coefficient and dissipated energy wear coefficient, $k_{E}/k_{A}$ is μ. Through Equation (4), the variation of wear coefficient can be considered, which was analyzed by Yue and Wahab [6].

**Figure 1:**
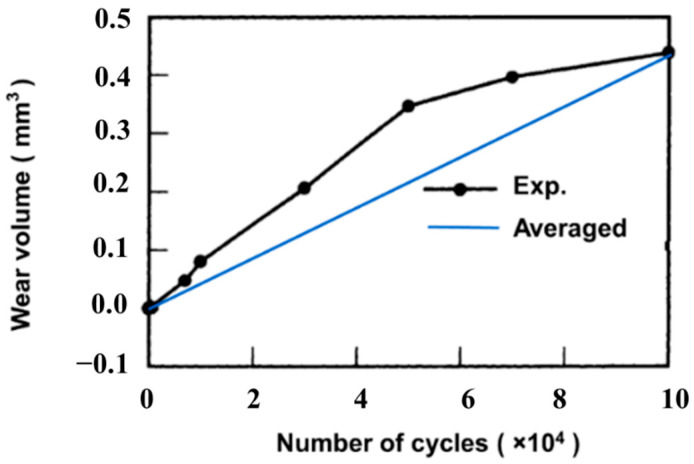
Experimental variation of wear volume with the number of cycles vs. assumed variation of wear volume in finite element (FE) model for different loading cases.

The wear coefficient in the above-mentioned two wear models is mostly considered as a constant in FE simulation, whereas the experimental results show the opposite. Figure 1 shows the experimental wear volume variation with the number of cycles, in which the slope of the experimental wear volume line is proportional to the wear coefficient based on Equation (2) [29,30]. The experimental setup is a ball-on-flat configuration. The corresponding loading conditions and materials are: normal load is 300 N, slip amplitude (half stroke) is 300 µm, and the ball’s radius is 30 mm. The slope of the blue line in Figure 1 is a constant averaged only through the final wear volumes based on Equation (2), which was used in most current research work. Through Figure 1, we can conclude that the wear coefficient varies with the number of cycles, which should be analyzed and considered in the FE model.

**Figure 2:**
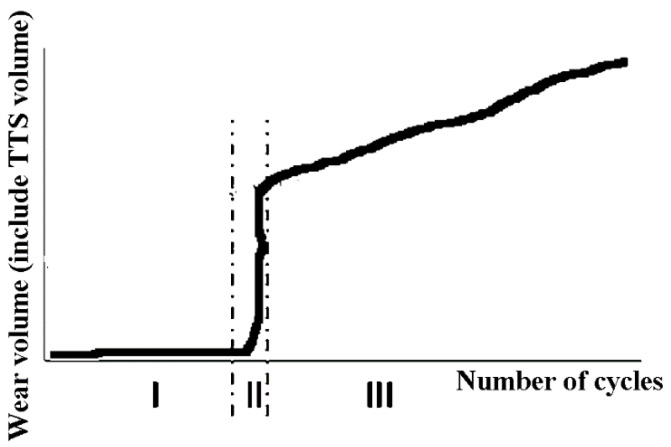
Three phases of the fretting wear process.

Zhou and Sauger proposed and analyzed TTS in the fretting process, which is the transition phase between the bulk material and debris [31,32,33]. The fretting wear process can be divided into three phases: (I) running in stage (plastic deformation accumulation); (II) TTS formation (TTS volume increases dramatically), (III) stable stage (the destruction of TTS and formation of debris) based on the experimental results, as shown in Figure 2 [30,34]. The debris formation begins at the beginning of stage III. In phase I and II, there is only plastic deformation accumulation and TTS formation, and the wear does not begin at this period, which is the TTS period. In most research, this phenomenon is ignored. The wear coefficient is commonly obtained by averaging the final cycle wear volume and is applied to all the fretting cycles in the FE model. It is interesting to consider the change in the analysis of variable wear coefficient during and after the running-in stage.

## 3. Experimental Setup

**Figure 3:**
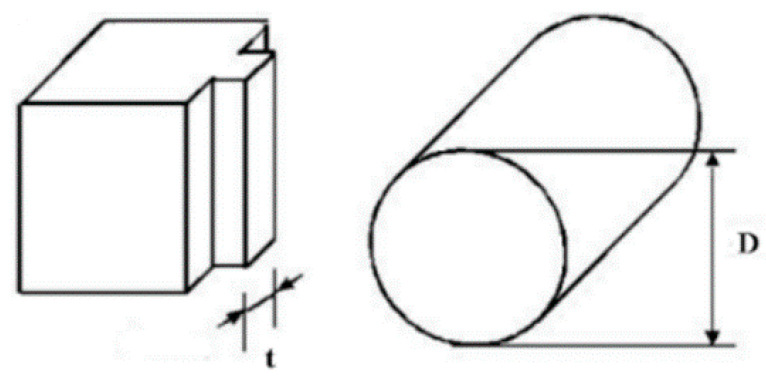
Geometry of the cylindrical pad and flat specimen.

**Figure 4:**
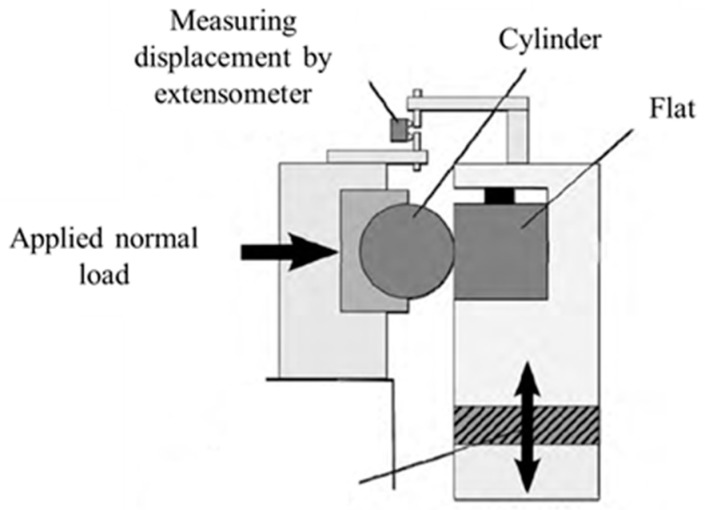
Sketch of the experimental setup.

The experimental results and setup are obtained from [30]. The geometry of the cylindrical pad and the flat specimen is shown in Figure 3, where the thickness of the flat specimen, t is 3 mm, and the diameter of cylindrical pad, D is 20 mm. The normal load, P is applied at the top of the cylindrical pad, and oscillatory displacement, δ (half stroke), is applied to the flat specimen. In this paper, P is 1000 N and δ is 50 µm in the gross sliding regime and 10 µm in the partial slip regime. The applied displacement is recorded by the extensometer. The sketch of the experimental setup is shown in Figure 4.

**Figure 5:**
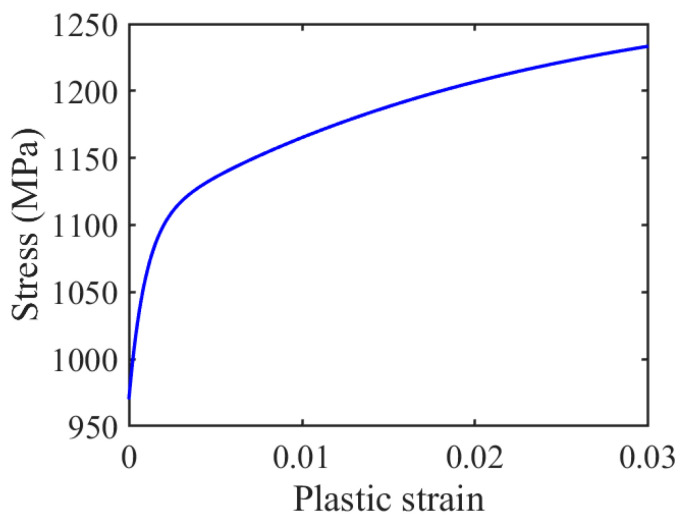
Relationship between plastic strain and stress.

The material properties are listed in Table 1. The coefficient of friction (COF) is 1.0 in the stable stage, as reported in ref. [30]. The tribology characteristics of the contact surfaces are only considered by COF in this paper. The plastic strain and stress curve is based on the equation in [35]:

(5) $$\sigma =\sigma_{y}+\frac{C_{1}}{\gamma_{1}}\left(1-e^{\gamma_{1}}\epsilon_{p}\right)+\;\frac{C_{2}}{\gamma_{2}}\left(1-e^{\gamma_{2}}\epsilon_{p}\right)$$

where $\sigma_{y}$ is the yield stress, and $C_{1}$, $\gamma_{1}$, $C_{2}$, and $\gamma_{2}$ are material property constants and determined by experiments and the corresponding values and units of these constants are 136,500, 1050, 8100 and 45. The obtained plastic stain and stress curve is plotted in Figure 5.

## 4. Numerical Models

### 4.1. FE Model

**Figure 6:**
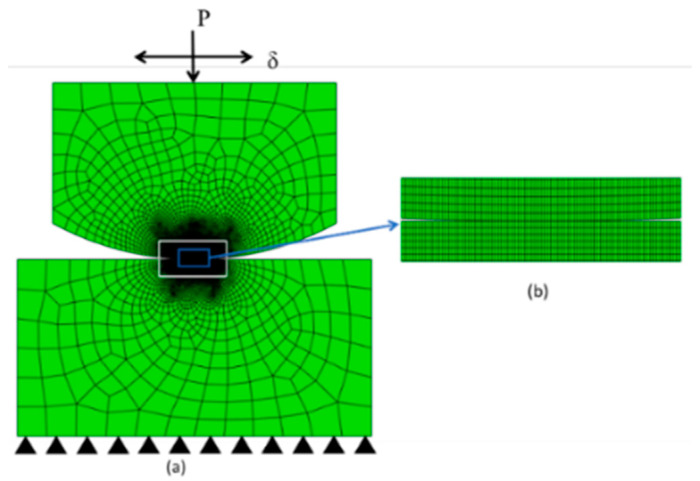
FE model of fretting wear, (**a**) globale view and (**b**) zoom at contact.

The nonlinear FE model is designed in the commercial software ABAQUS by Python scripts, shown in Figure 6a. 4-node bilinear plane strain quadrilateral (CPE4) element is applied to the whole model. Lagrange multiplier is used to solve the contact. Normal behavior is set as hard contact, while the tangential behavior is set as Coulomb’s friction law with isotropic friction. The radius of the cylindrical pad is 10 mm. To consider both efficiency and accuracy of the model, the partition is used for both parts. The mesh in both contact zones is shown in Figure 6b, and the mesh size is 10 µm × 20 µm.

**Figure 7:**
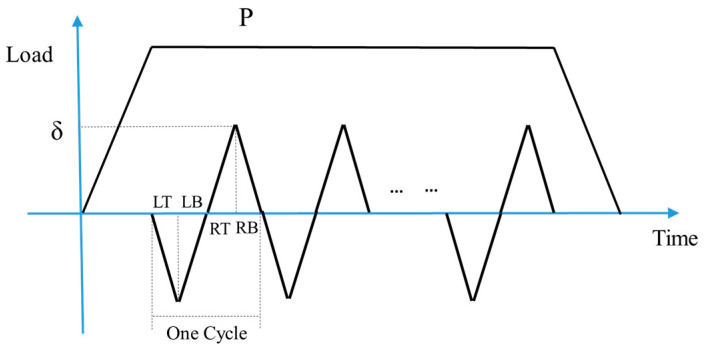
Loading history of FE model.

**Figure 8:**
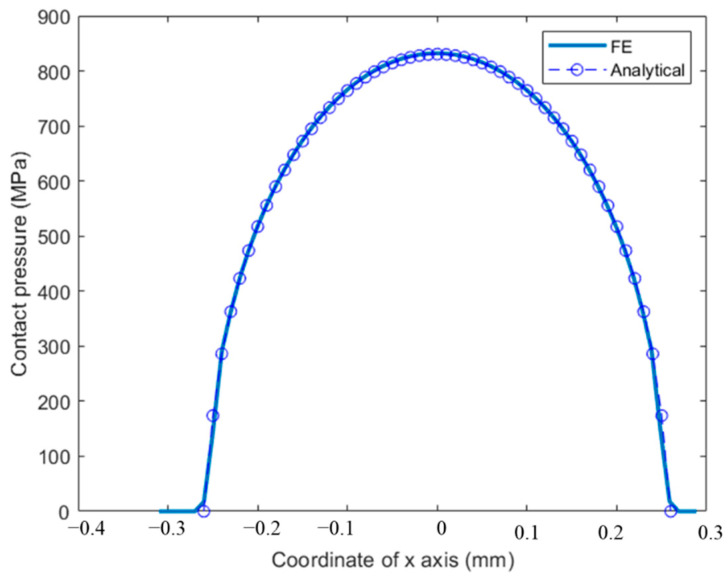
Contact pressure comparison between the FE model and analytical solution when *P* = 1000 N.

The normal load and slip between the two contact parts are both relatives. To simplify the model, we apply the normal load and oscillatory displacement on the top center of the cylindrical pad. The loading history is shown in Figure 7. In one cycle, there are four steps: LT, LB, RT, RB (as indicated in Figure 7), which represent the different tangential positions of the flat. δ is the applied tangential displacement, and before the oscillatory relative slip, normal load, P is applied. Moreover, after all the cycles, there is a releasing step for the normal load. To save more computation time, jump cycle, ΔN is used, which means that after each increment, the wear depth is obtained by multiply ΔN in Equation (4). The first 2500 cycles are the TTS period, and the wear coefficient is set as 0 in this stage. The dissipated energy wear equation was used in the user subroutine, UMESHMOTION. To validate the FE model, the contact pressures of both the FE model and analytical solution are compared, as shown in Figure 8.

### 4.2. Modeling the Variation in Wear Coefficient

**Figure 9:**
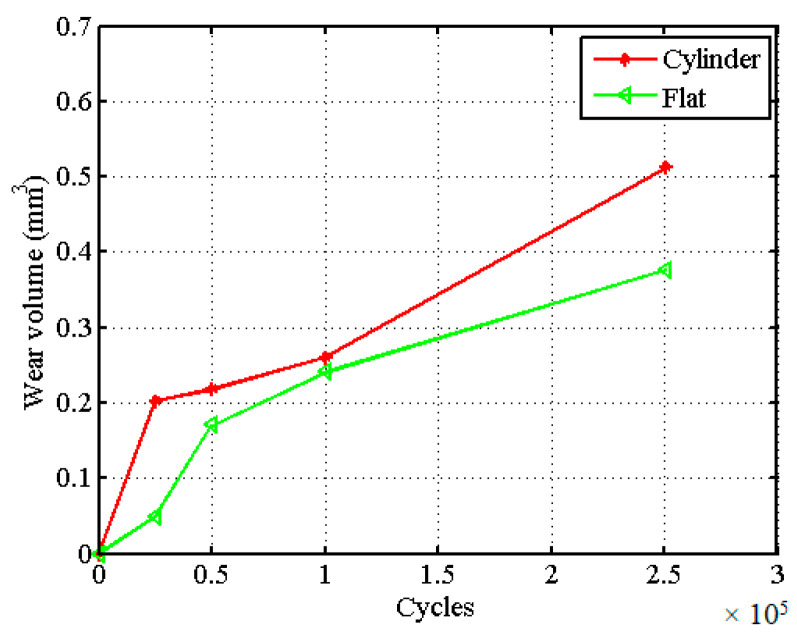
Wear coefficient changes with the number of cycles for both flat specimen and cylindrical pad.

The variation of wear coefficient during the fretting process is an interesting topic. As shown in Figure 9, the wear coefficient of both the cylindrical pad and the flat specimen varies with the number of cycles [30]. In this paper, two wear coefficient models are used, namely the constant wear coefficient model (CWCM) and the variable wear coefficient model (VWCM). To consider this effect in the partial slip regime, the wear coefficient is taken the same value as that in the gross sliding regime. By averaging each line in Figure 9, the Archard’s wear coefficient variation with a certain cycle’s period for both cylinder and flat is shown in Figure 9, which is used for VWCM. For CWCM, the averaged wear coefficient is obtained through the wear volume at the 25,000th cycle.

**Figure 10:**
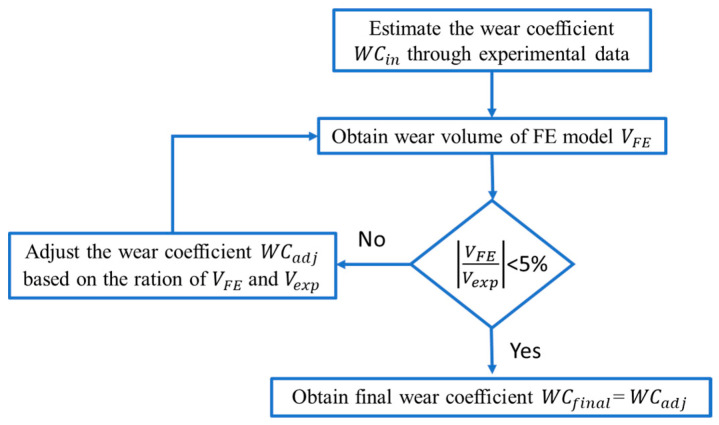
Flowchart to obtain the wear coefficient to be used in the FE model.

**Figure 11:**
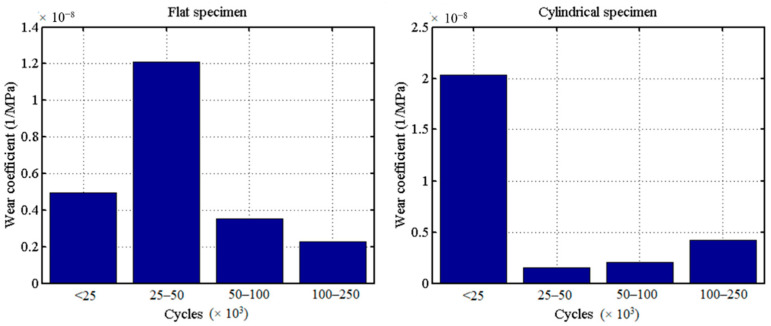
Experimental wear coefficient in certain cycles’ interval: (**a**) flat specimen, (**b**) cylindrical pad.

To obtain the wear coefficient for every cycle period of the flat and cylindrical pad, relative slip amplitude on the contact surface, δ should be known at first. Based on Equation (1), the estimated wear coefficient is 9.98 × 10^−9^ MPa^−1^ in the 25,000 cycles for flat. To make the wear coefficient more accurate to be used in the FE model, a wear coefficient modification method is applied in this paper, as is shown in Figure 10 [36]. From the figure, we can see that based on the estimated wear coefficient, the wear volume of FE is compared with the wear volume of the experiment until it meets a certain accuracy by adjusting the wear coefficient step-by-step. The wear coefficients for all the periods in Figure 9 are shown in Figure 11.

From Figure 11, we can see that the wear coefficient is not constant in the whole fretting process. Due to the limitation of the experimental data, we can only get the wear coefficient in a certain period. We do not know where there is a variation of wear coefficient in four cycles’ intervals. We take the first 25,000 cycles of the flat specimen as an example: The averaged wear coefficient is much lower in the first 25,000 cycles compared with that in 25,000–50,000 cycles. This means that in the first 25,000 cycles, there is a certain point after which the wear coefficient increased dramatically. The opposite phenomenon happens for the cylindrical pad: In the first 25,000 cycles, there is a certain value, after which there is a significant decrease in wear coefficient. Moreover, based on the experimental data of the COF [30], the first 2500 cycles are considered a TTS period, which means that the wear coefficient is 0 in this period. The averaged Archard’s wear coefficients for flat and cylinder are 4.99 × 10^−9^ and 2.058 × 10^−8^ MPa^−1^ in the first 25,000 cycles, respectively.

**Figure 12:**
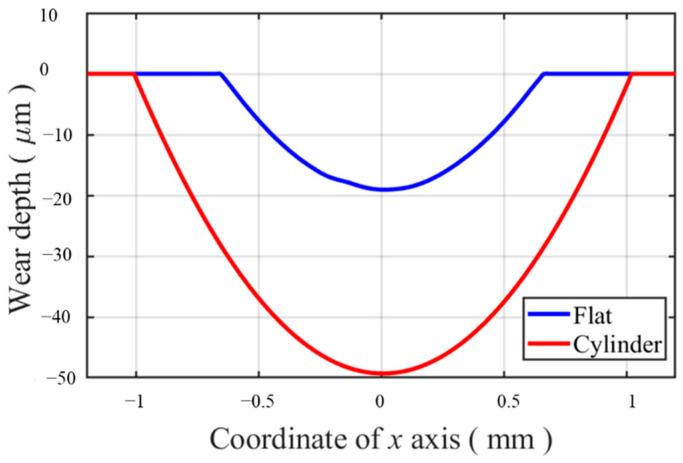
Wear characteristics of both specimens after 25,000 cycles.

Validation of the wear models is applied for both specimens. The cross-sections of both specimens are shown in Figure 12. After integrating the wear profile, the thickness is used to multiply the integration results to obtain the computational wear volume. The computational wear volumes are 0.0479 and 0.1998 mm^3^ for flat and cylindrical pads by CWCM after 25,000 cycles, respectively. Compared with the experimental result, 0.0497 and 0.2026 mm^3^, we can see that the computational wear characteristics by CWCM show a good agreement with experimental results.

**Figure 13:**
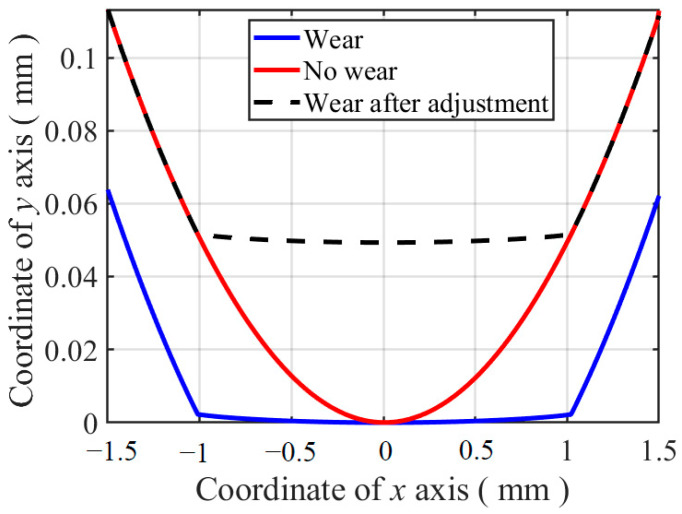
Profiles of the cylinder before and after fretting wear and the adjusted wear profile.

In the FE model, the wear profile of the flat specimen can be obtained by export the coordinate of contact surface nodes after the fretting wear process directly. However, for the cylindrical pad, it is not the same case. The profiles of the cylinder before and after fretting wear are shown in Figure 13. Due to the wear on the cylinder contact surface, there is an overall movement. To make the wear profile more comparable, adjustment is applied to make the profiles agree with each other, wherever there is no wear on it. The difference between the profiles with wear and without wear is the wear profile of the cylinder obtained in Figure 12.

**Figure 14:**
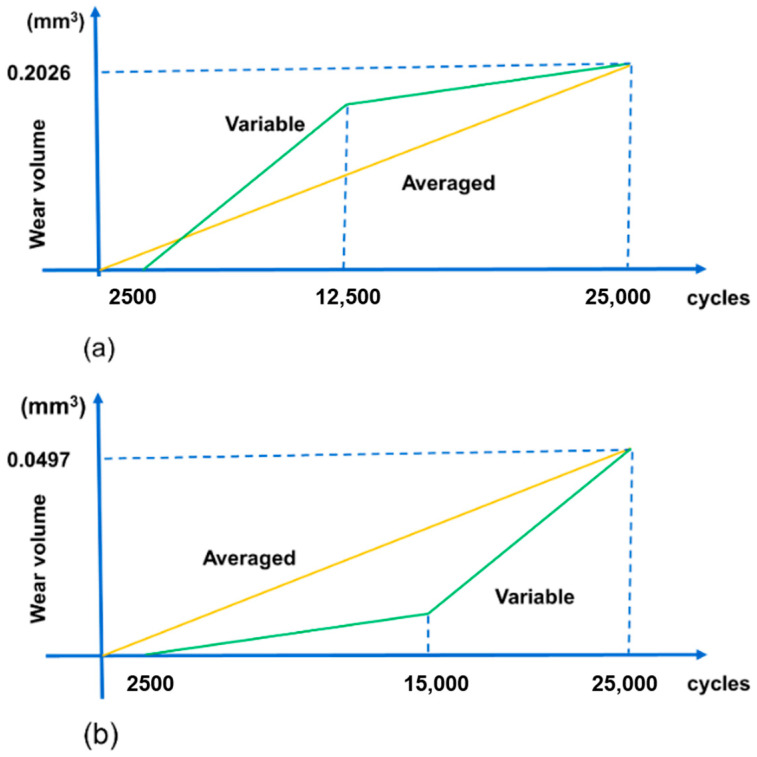
Schematic of constant wear coefficient model (CWCM) and variable wear coefficient model (VWCM) in the first 25,000 cycles for both specimens: (**a**) cylindrical pad; (**b**) flat specimen.

Considering the above conditions, double linear models are used for both flat specimen and cylindrical pad in the first 25,000 cycles. The wear volume after 25,000 cycles is set to be the same as the experimental data for both specimens. CWCM and VWCM in the first 25,000 cycles for both specimens are shown in Figure 14.

In Figure 14, we assumed that the slop of the sharp increment is 5 times greater than that of the moderate growth and the sharp increment period is 10,000 cycles, while the moderate growth period is 12,500 cycles for both cylindrical pad and flat specimen. Based on Equation (2), two variable Arcahrd’s wear coefficients, $k_{A}$ are 1.996 × 10^−9^ and 9.98 × 10^−9^ for flat specimen and are 8.23 × 10^−9^ and 4.115 × 10^−8^ for the cylindrical pad. Because COF is 1.0 in the stable stage, the dissipated energy wear coefficient $k_{E}$ is the same as $k_{A}$.

### 4.3. Numerical Implementation

**Figure 15:**
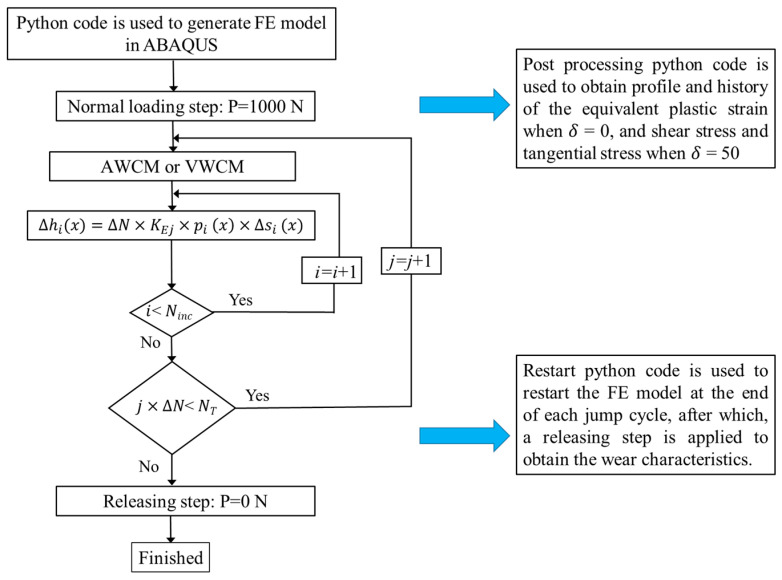
Flowchart of numerical implementation for cylindrical pad and flat specimen.

Flowchart of the numerical implementation for the cylindrical pad and the flat specimen is shown in Figure 15, where $N_{inc}$ is the total number of increment; $K_{Ej}$ is the wear coefficient, which changes with the number of cycles and is obtained based on Figure 14 for both CWCM and VWCM, and $N_{T}$ is the total number of cycles. From the figure, we can see that after obtaining the simulation result of CWCM and VWCM for both specimens, postprocessing and restart Python code are used to get the corresponding parameters’ history and profile. To obtain the wear characteristics history, a releasing step should be created at the end of each jump cycle by restarting, and the former steps after this jump cycle are deleted in the restarted CAE model.

As described in [27], the stability of the model is related to the local wear depth after each increment in the FE simulation process. The local wear depth is proportional to the jump cycles. Based on Figure 11 and Figure 14, we can see that the cylinder’s wear coefficient is much greater than that of the flat specimen. Therefore, to make the simulation process more stable, 1250 cycles and 625 cycles are used as jump cycles for flat and cylinder parts by trial and error, respectively.

## 5. Results and Discussion

The corresponding wear characteristics, plastic strains and stresses, for the gross sliding regime and partial slip regime are analyzed in detail in the following two sections, respectively.

### 5.1. Gross Sliding Regime

#### 5.1.1. Flat Specimen

**Figure 16:**
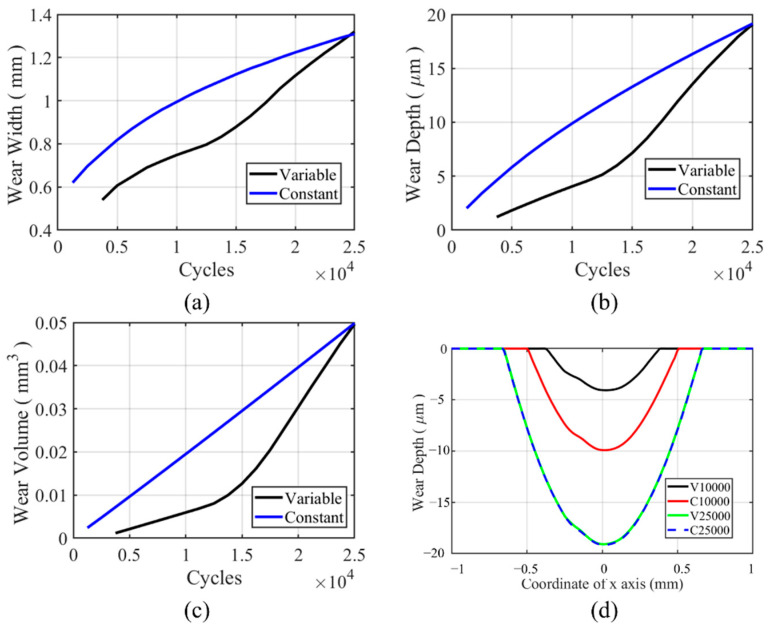
History and profile of wear characteristics for the flat specimen in the gross sliding regime for both CWCM and VWCM: (**a**–**c**) are the history of wear characteristics, and (**d**) is the wear profiles after 10,000th and 25,000th cycle.

For flat specimens, a moderate-sharp VWCM and CWCM are used. The wear characteristics history can be found in Figure 16a–c. From the figure, we can see that the trend for wear depth, wear width, and wear volume history is the same. In the moderate increment period, the gap between the wear characteristics tends to be greater, while in the sharp increment period, the gap between wear characteristics for VWCM and CWCM tends to be lower. At the end of the 25,000th cycle, there is no significant difference for all the wear characteristics. From Figure 16d, we can see that in the moderate increment period, i.e., 10,000th cycle, the wear width and wear depth are both greater in CWCM compared with that in VWCM, and at the end of the 25,000th cycle, the wear profile of VWCM shows a good agreement with that of CWCM. Moreover, there are some ripples on the left side of the wear profile for both CWCM and VWCM, which are caused by plastic deformation.

**Figure 17:**
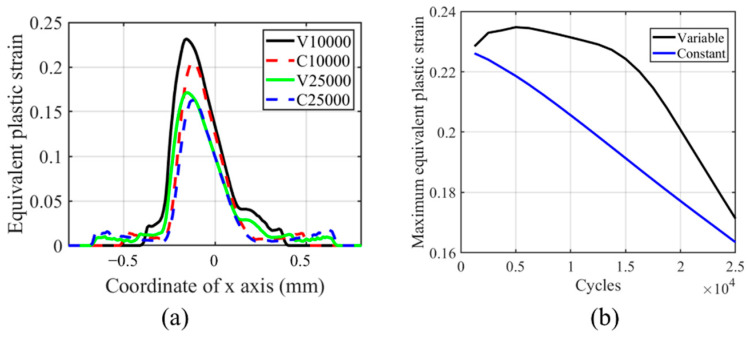
Profile and history of equivalent plastic strain for the flat specimen in the gross sliding regime for both CWCM and VWCM when δ = 50 mm: (**a**) the profiles at 10,000th cycle and 250,000th cycle, and (**b**) the maximum value history.

From Figure 17, we can see that the maximum equivalent plastic strain is located on the left-hand side of the contact center, and the maximum value decreases with the number of cycles. The offset is caused by the initial movement direction of the cylinder. In the initial left movement of the cylinder, the von Mises stress decreases on the contact surface due to the plastic deformation. Therefore, the peak value cannot be reached again when the cylinder moves to the far-right side of contact unless the relative slip amplitude is much greater than the contact zone. This phenomenon is explained in [37,38]. Another interesting phenomenon is that the plasticity decreases with the number of cycles, and the plastic strain on both tailing and leading edges increases with the number of cycles. This phenomenon is caused by the evolution of the wear profile, which removes the residual plastic strain [36]. Moreover, there is a stress concentration on both trailing and leading edges, which causes plastic deformation. From Figure 17b, we can see that after an increase of the maximum value of equivalent plastic strain in the TTS period, there is a smooth decrease in the peak value. This means that in the moderate increment period, the equivalent plastic strain decreases smoothly, while in the sharp increment period, the equivalent plastic strain decreases dramatically. Due to the increase in the TTS period and the moderate decrease in the moderate increment period, the maximum equivalent plastic strain is always higher in VWCM than that in CWCM.

**Figure 18:**
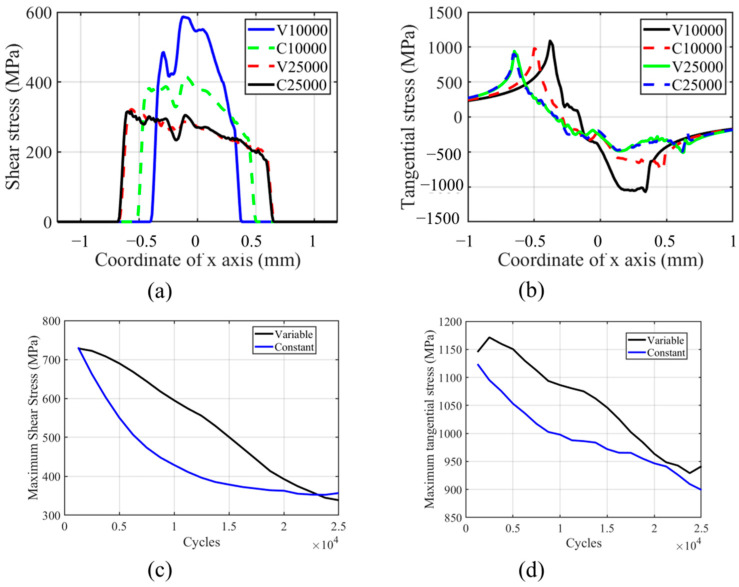
Profile and history of shear and tangential stresses for the flat specimen in the gross sliding regime for both CWCM and VWCM: (**a**,**b**) are shear and tangential stress profiles in 10,000th cycle and 250,000th cycle when δ = 50 mm, and (**c**,**d**) are the history of maximum shear and tangential stress, respectively.

The shear stress and tangential stress history in the fretting process are shown in Figure 18. Both shear and tangential stress are decreasing with the number of cycles due to the wear profile evolution. The tangential and shear stress profiles are not smooth on the left-hand side of the contact center, which is caused by the plastic deformation in this zone. Similar to other research results in the literature, the contact zone increases with the number of cycles, as shown in Figure 18a [27]. Moreover, due to the lower wear coefficient in the moderate increment period, the contact pressure of VWCM in the 10,000th cycle is greater than that of CWCM in the 10,000th cycle. From Figure 18b, we can see that the maximum tangential stress location moves toward the left contact edge with the number of cycles. Moreover, in the TTS period, the tangential stress increased with the number of cycles, after which it decreases smoothly with the number of cycles. From Figure 18c,d, we can see that the tangential and shear stresses are both greater in VWCM than that in CWCM during almost the whole fretting process.

#### 5.1.2. Cylindrical Pad

**Figure 19:**
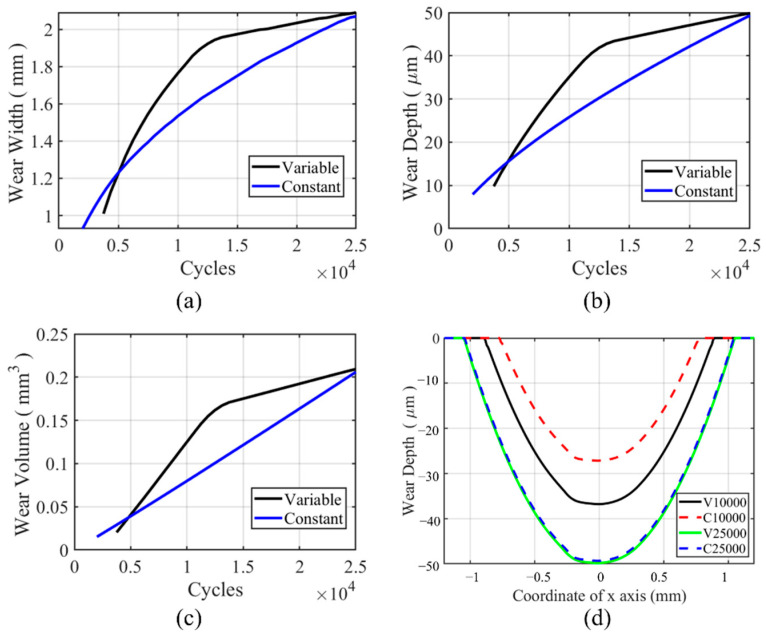
History and profile of wear characteristics for the cylindrical pad in the gross sliding regime for both CWCM and VWCM: (**a**–**c**) are the history of wear characteristics, and (**d**) is the wear profiles after the 10,000th and 25,000th cycle.

For the cylindrical pad, a sharp-moderate VWCM and CWCM are used. The wear characteristics in VWCM, wear width, wear depth, and wear volume are lower in the TTS period and then increase dramatically in the sharp increment period, after which wear characteristics in VWCM exceed that in CWCM at about the 5000th cycle. At the end of the 25,000th cycle, the wear characteristics of VWCM and CWCM show a good agreement with each other. The ripples on the left-hand side also happened to the cylindrical pad due to the plastic deformation. Based on Figure 16 and Figure 19, we can see that in the gross slip regime, the variable wear coefficient has no significant effect on the wear characteristics.

**Figure 20:**
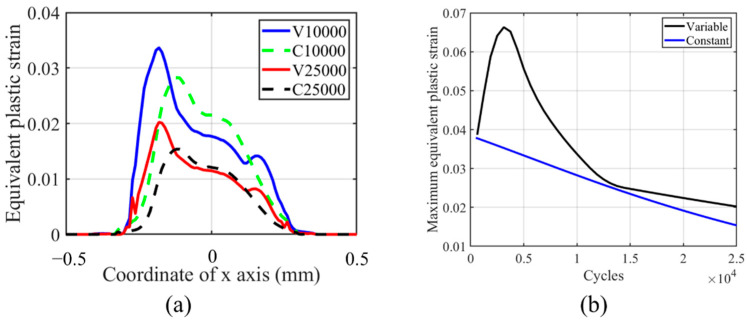
Profile and history of the equivalent plastic strain for the cylindrical pad in the gross sliding regime for both CWCM and VWCM when δ = 50 mm: (**a**) is the profiles in 10,000th cycle and 250,000th cycle, and (**b**) is the maximum value history.

The same trend is shown in Figure 20 as in Figure 17. The peaks of equivalent plastic strain locate on the left-hand side of the contact zone and decrease with the number of cycles. The peak value in VWCM is greater than that in CWCM, which is caused by high plastic strain in the TTS period. Based on Figure 17 and Figure 20, we can conclude that in the gross sliding regime, there is an increase of equivalent plastic strain in the TTS period, and a higher wear coefficient can remove the residual plastic strain faster.

**Figure 21:**
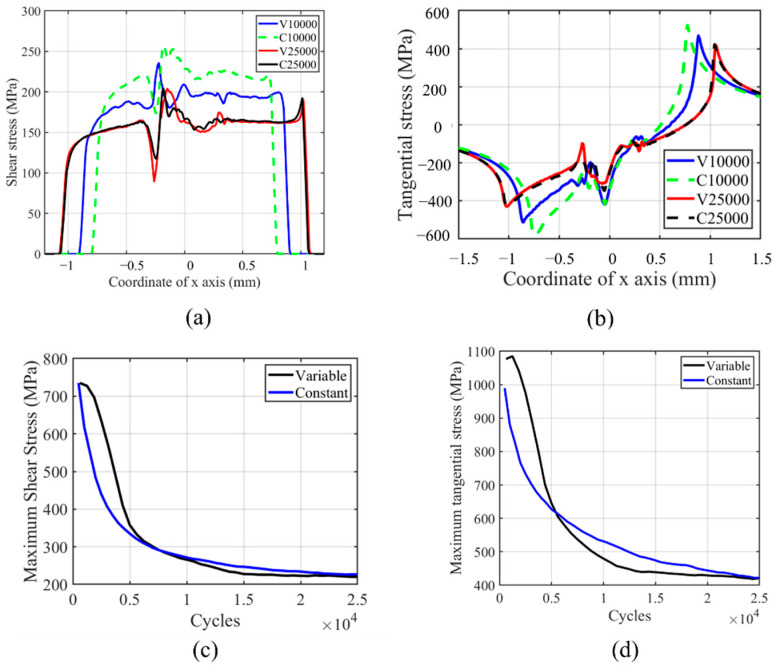
Profile and history of shear and tangential stress for the cylindrical pad in the gross sliding regime for both CWCM and VWCM: (**a**,**b**) are shear and tangential stress profiles in 10,000th cycle and 250,000th cycle when δ = 50 mm, and (**c**,**d**) are the history of maximum shear and tangential stress, respectively.

The tangential and shear stress profiles and maximum value history are shown in Figure 21. From Figure 21c,d, we can see that after the TTS period, the maximum value of both tangential and shear stress decreased dramatically in the sharp increment period, after which the maximum value is lower in VWCM than that in CWCM. Moreover, in the moderate increment period, the trend tends to be smooth. The peak values for both stresses tend to be the same for both VWCM and CWCM at the end of the 25,000th cycle. From Figure 21a,b, we can see that the position of maximum shear stress is near the maximum equivalent plastic strain point, while the maximum and minimum tangential stresses are located near the contact edge on both sides, respectively. Based on Figure 18 and Figure 21, we can see that in the gross sliding regime, the effect of variable wear coefficient on shear and tangential stress is the same as that on the equivalent plastic strain.

### 5.2. Partial Slip Regime

The wear coefficient in the partial slip regime is assumed to be the same as that in the gross sliding regime. Therefore, the applied displacement D = 12 µm is used to investigate the characteristics history, stress condition in the partial slip regime.

#### 5.2.1. Flat Specimen

**Figure 22:**
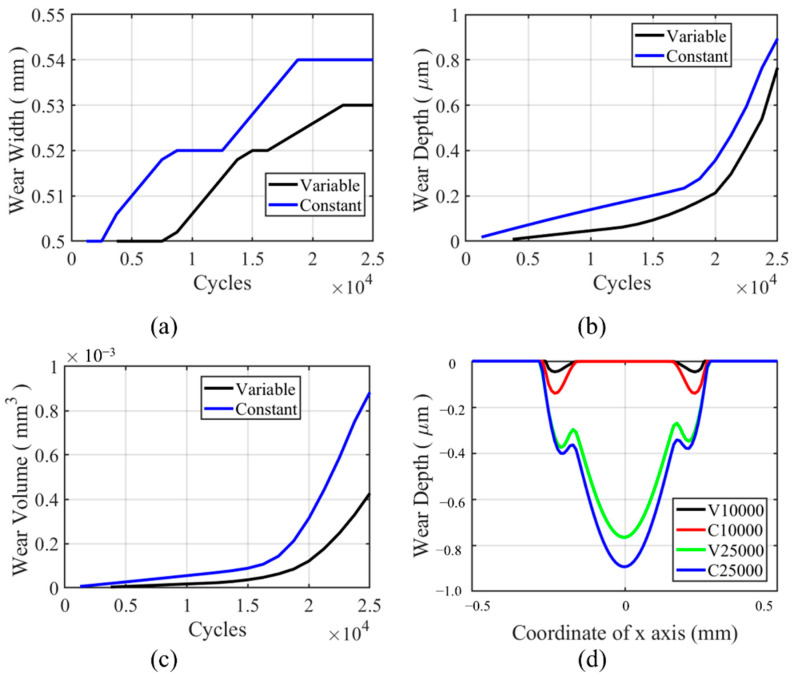
History and profile of wear characteristics for the flat specimen in the partial slip regime for both CWCM and VWCM: (**a**–**c**) are the history of wear characteristics, and (**d**) is the wear profiles after 10,000th and 25,000th cycle.

**Figure 23:**
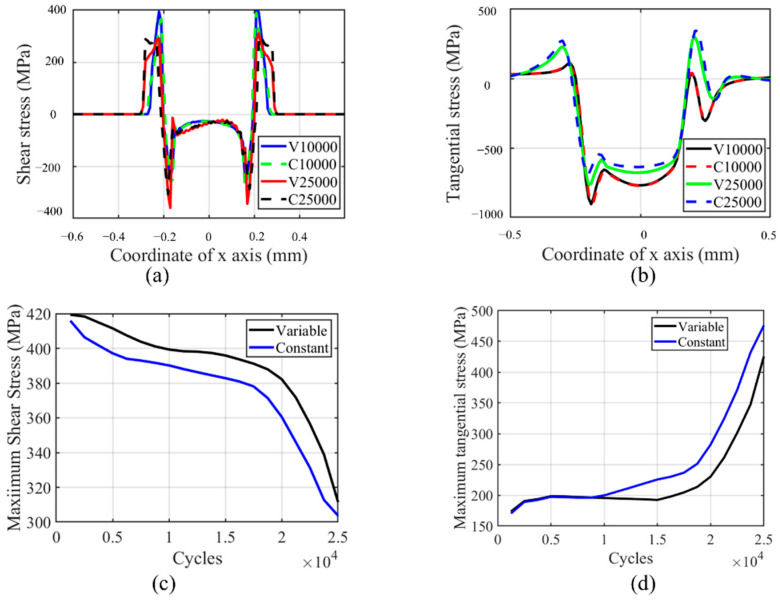
Profile and history of shear and tangential stress for the flat specimen in the partial slip regime for both CWCM and VWCM: (**a**,**b**) are shear and tangential stress profiles in 10,000th cycle and 250,000th cycle when δ = 10 mm, and (**c**,**d**) are the history of maximum shear and tangential stress, respectively.

The wear characteristic for the flat specimen in the partial slip regime is shown in Figure 22. From Figure 22a, we can see that the wear width in VWCM is lower than that in CWCM in the whole process. The line for wear width is not smooth due to the mesh size. For Figure 22b,c, we can see that the wear depth and wear volume in VWCM are also lower than that in CWCM in the whole fretting process. From Figure 22d, due to the wear in the partial slip zone, the slip happens in the stick zone to accommodate the applied displacement for both VWCM and CWCM. There is a dramatic increase in wear depth and wear volume after the 20,000th cycle due to this phenomenon. Because the shear stress decreases dramatically with cycles, as is shown in Figure 23c, the higher shear stress can cause more wear volume with a higher wear coefficient before the 15,000th cycle and then the time point when the stick zone slips for CWCM is earlier than that for VWCM, after which the wear volume increased dramatically. The transition time point is about 17,500th cycle for CWCM, while for VWCM, the transition time point is about 20,000th cycle.

**Figure 24:**
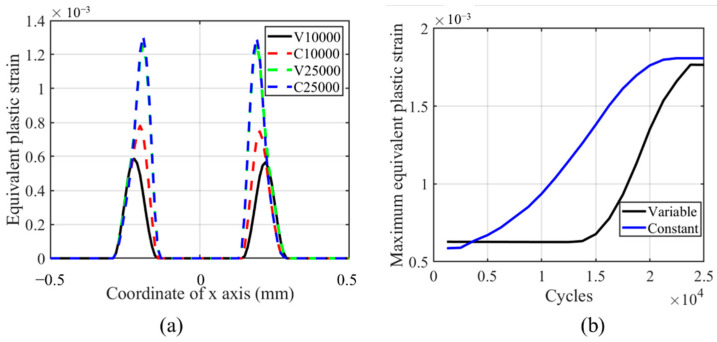
Profile and history of equivalent plastic strain for the flat specimen in the partial slip regime for both CWCM and VWCM when δ = 10 mm: (**a**) is the profiles in 10,000th cycle and 250,000th cycle, and (**b**) is the maximum value history.

From Figure 24, we can see that the accumulation of plasticity increases in the partial slip regime with the number of cycles, while in Figure 17, the trend is the opposite. This phenomenon is caused by the lower wear effect in the partial slip regime on plasticity accumulation. The maximum and profile of plastic strain show no significant difference between VWCM and CWCM. The maximum plastic strain locates near the initial contact edge. Due to the lower wear coefficient for flat specimens and lower relative slip in the partial slip regime, the effect of the TTS period on the accumulation of plasticity in VWCM is not significant compared with that in CWCM. At the end of the 25,000th cycle, the maximum equivalent strain in CWCM is a little greater than that in VWCM, which is caused by stable plastic accumulation in CWCM. The effect of wear on plastic accumulation is kind of positive for flat in partial slip regime, which is caused by the stress concentration in the wear process.

The tangential and shear stress profiles and maximum value history are shown in Figure 23 for a flat specimen in a partial slip regime. From Figure 23a,b, we can see that the maximum shear stress decreases, while the minimum shear stress increases with the number of cycles. Maximum tangential stress and minimum tangential stress show the opposite trend. Through the history of the maximum shear and tangential stress shown in Figure 23c,d, we can see that the effect of the wear characteristics on the maximum value of tangential and shear stress is the opposite. The maximum tangential stress history shows a similar trend with wear characteristics’ history, while the maximum shear stress history shows the opposite trend.

#### 5.2.2. Cylindrical Pad

**Figure 25:**
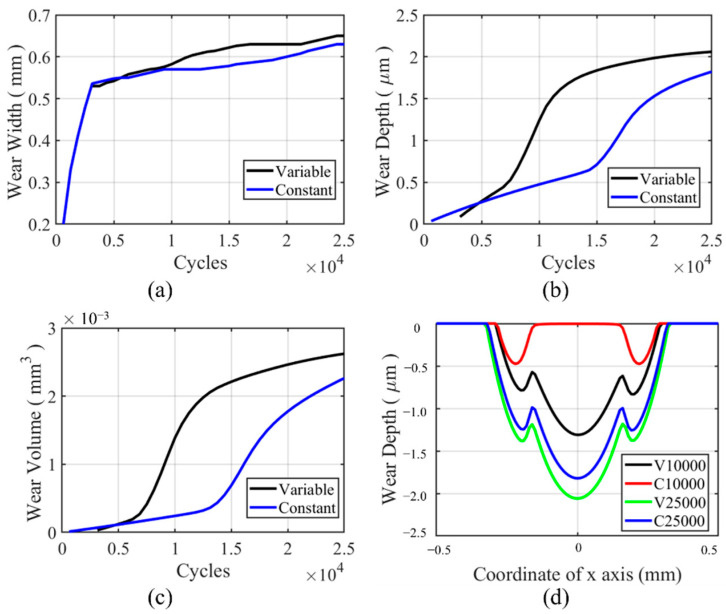
History and profile of wear characteristics for the cylindrical pad in the partial slip regime for both CWCM and VWCM: (**a**–**c**) are the history of wear characteristics, and (**d**) is the wear profiles after the 10,000th and 25,000th cycle.

Wear characteristics for the cylindrical pad in partial slip regime are shown in Figure 25. From Figure 25a–c, we can see that after the TTS period, there is a dramatic increment of wear width, wear depth and wear volume in VWCM in the sharp increment period and then smooth increment in the moderate increment period. The wear depth and wear volume in VWCM are both higher after about the 6000th cycle than that in CWCM. Though the wear width is almost the same at the 25,000th cycle, the wear depth and wear volume are greater in VWCM than in CWCM. The wear coefficient of the cylindrical pad is much higher than that of the flat specimen. The stick zone begins to slip after about the 6000th cycle with a higher wear coefficient and 15,000th cycle with a constant wear coefficient, respectively, as shown t in Figure 25b,c. Therefore, the stick zone begins to slip earlier and with a higher wear coefficient in VWCM than that in CWCM, which causes more wear in the initial stick zone in VWCM with higher contact pressure as is shown in Figure 25c and makes the wear depth, wear width, and width higher in VWCM at the end of the 25,000th cycle.

**Figure 26:**
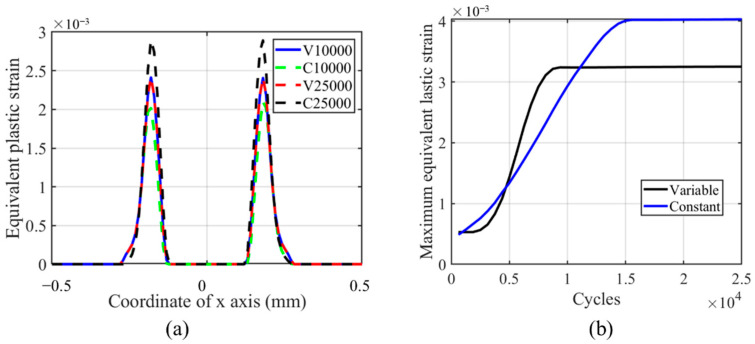
Profile and history of the equivalent plastic strain for the cylindrical pad in partial slip regime for both CWCM and VWCM when δ = 10 mm: (**a**) is the profiles in 10,000th cycle and 250,000th cycle, and (**b**) is the maximum value history.

From Figure 26, the equivalent plastic strain increases with the number of cycles for the cylindrical pad in the partial slip regime, which shows the same trend for the flat specimen in the partial slip regime. The maximum value in CWCM is lower than that in VWCM at the 10,000th cycle and is greater than that in VWCM at the 25,000th cycle. Moreover, due to more material removal in VWCM, the equivalent plastic strain in CWCM exceeds that in VWCM, as shown in Figure 25d. The maximum equivalent plastic strain tends to be stable after the slipping of the initial stick zone.

**Figure 27:**
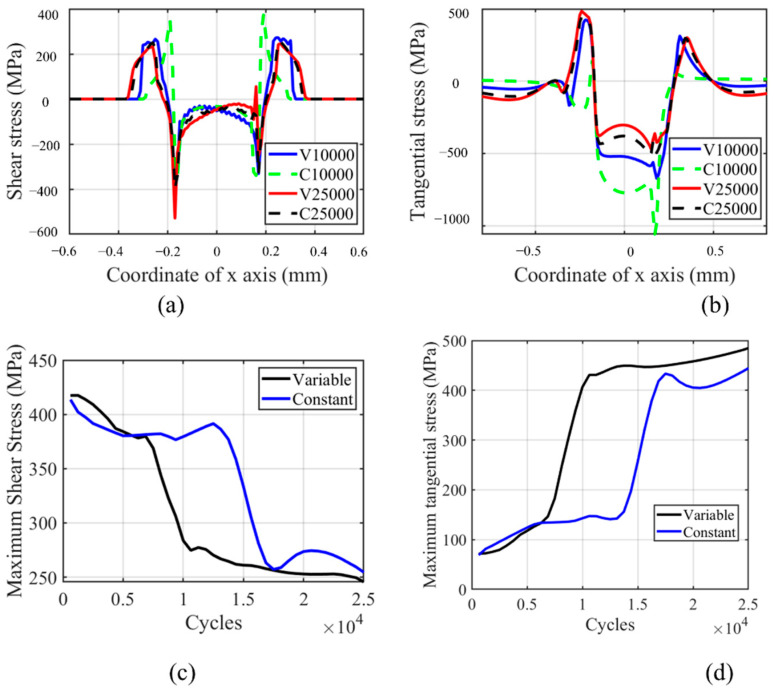
Profile and history of shear and tangential stress for a cylindrical pad in the partial slip regime for both CWCM and VWCM: (**a**,**b**) are shear and tangential stress profiles in 10,000th cycle and 250,000th cycle when δ = 10 mm, and (**c**,**d**) is the history of maximum shear and tangential stress, respectively.

The history and profile of tangential and shear stress are shown in Figure 27. From Figure 27a,b, we can see that the maximum tangential and shear stresses are located near the contact edge and move far away from the contact center with the number of cycles. From Figure 27c,d, we can see that the trend of shear and tangential stresses is opposite with the number of cycles. The dramatic decrease of shear stress and increase of tangential stress happen near the cycle in which the initial stick zone begins to slip for both CWCM and VWCM. At the 25,000th cycle, the maximum shear stress is lower, and the tangential stress is greater in VWCM than that in CWCM, which shows the same trend as for wear depth and volume in Figure 23c,d.

## 6. Conclusions

In a gross sliding regime, the characteristics of the wear profile have an opposite effect on the maximum tangential and shear stress for both flat specimen and cylindrical specimen. Lower maximum stress corresponds to the higher wear characteristics. The effect of wear volume on the plasticity accumulation is significant. The higher wear volume caused less plasticity accumulation. The effect of VWCM for flat and cylindrical specimens on the history of stress, wear characteristics and plasticity accumulation are significant.

In a partial slip regime, the characteristics of the wear profile have an opposite effect on the maximum shear stress and a positive effect on the tangential for both flat specimen and cylindrical specimen. Lower maximum shear stress and higher maximum tangential stress corresponds to the higher wear characteristics. The effect of wear volume on the plasticity accumulation is not significant. Plasticity accumulation in CWCM tends to be higher than that in VCWM for both specimen and cylinder. The effect of VWCM for flat and cylindrical specimens on the history of stress, wear characteristics and plasticity accumulation are significant.

The history of stress and strain in VWCM differs from that in CWCM. Stress and strain history can affect the crack initiation lifetime. Therefore, the effect of VWCM on crack initiation can be future investigated.

## Figures and Tables

**Table 1:** Material properties.

**Young’s Modulus (GPa)**	119
**Poisson Ratio**	0.29
**Yield sStress (MPa)**	970

## Data Availability

Data sharing not applicable.
